# Identification of common diagnostic genes and molecular pathways in endometriosis and systemic lupus erythematosus by machine learning approach and in vitro experiment

**DOI:** 10.7150/ijms.101754

**Published:** 2025-01-01

**Authors:** Pusheng Yang, Yiping Zhu, Yaxin Miao, Tao Wang, Wenwen Liu, Jiaxin Zhang, Beilei Ge, Jing Sun

**Affiliations:** Shanghai Key Laboratory of Maternal Fetal Medicine, Shanghai Institute of Maternal-Fetal Medicine and Gynecologic Oncology, Shanghai First Maternity and Infant Hospital, School of Medicine, Tongji University, Shanghai 200092, China.

**Keywords:** inflammatory diseases, endometriosis, systemic lupus erythematosus, co-diagnostic genes, personalized medicine

## Abstract

Growing research suggests that endometriosis and systemic lupus erythematosus (SLE) are both chronic inflammatory diseases and closely related, but no studies have explored their common molecular characteristics and underlying mechanisms. Based on GEO datasets, differentially expressed genes in the endometriosis cohort and the SLE cohort were screened using Limma and weighted gene co-expression network analysis (WGCNA), and prediction signatures were constructed using LASSO logistic regression analysis, respectively. Four co-diagnostic genes (PMP22, QSOX1, REV3L, SP110) were identified for endometriosis and SLE. The nomogram, calibration curve, decision curve analyses (DCA), area under the receiver operating characteristic (AUC) curve and external datasets were used to evaluate the diagnostic and predictive value of co-diagnostic genes. The AUC value of the four co-diagnostic genes were higher than 0.85 in both endometriosis and SLE cohorts. Besides, functional enrichment analysis showed that DNA replication, base excision repair, cell cycle and cell adhesion molecules were significantly enriched. Multifactor regulatory network of four co-diagnostic genes was constructed including 96 TFs, 42 miRNA, 43 lncRNA, and 189 drugs, and Tributyrin was found to act on four co-diagnostic genes simultaneously. We identified and validated four co-diagnostic genes and revealed the potential molecular mechanisms of endometriosis and SLE, which is helpful for early diagnosis and targeted therapy. Our study provides a novel perspective for individualized treatment of patients with endometriosis and SLE.

## Introduction

Endometriosis, an estrogen-dependent and chronic inflammatory gynecological disease, is characterized by the viable endometrial tissues survived outside the uterine cavity, resulting in dysmenorrhea, chronic pelvic inflammation and even infertility, thus affecting about 10% of reproductive-aged women 1. The underlying pathogenesis of endometriosis is complex and uncertain, but an increasing amount of evidence indicated that intrinsic disorder in endometrial stromal cells and abnormalities in immune system may contribute to its progression 2, 3. It has been reported that endometriosis patients had significant alterations in cellular and humoral immune responses, such as aberrant activation of lymphocyte, increased cytotoxicity and number of macrophages, decreased activity of natural killer (NK) cells, massive release of inflammatory factors, and altered autoantibodies 4, 5. In addition, endometriosis exhibited many similarities with autoimmune diseases, including tissue damage, abnormal T- and B-cell activation, autoantibodies production (against endometrium and phospholipids), recurrent immune-mediated abortion, and association with other autoimmune diseases 6. Therefore, it is essential to explore the molecular features and mechanisms involved in the progression of endometriosis from an immunological perspective to provide new strategies for the diagnosis and treatment of endometriosis.

Systemic lupus erythematosus (SLE) is a chronic autoimmune inflammatory disease characterized by abnormal activity of the immune system, including production of antinuclear antibodies, overreaction of B cells and T cells, deposition of immune complexes, and excessive activation of complement as well as cytokines 7. Patients with SLE exhibit immune-mediated inflammatory damage in virtually every organ system (such as the skin, kidneys, joints, and cardiovascular), predominantly in females (9:1) and manifesting during the childbearing years, often resulting in poor reproductive and obstetric outcomes 8. Several research suggested that estrogen hormone was strongly associated with increased incidence and progression of SLE in women 9. However, further investigation is required into the association between the SLE and gynecological diseases.

Accumulated evidence indicated that endometriosis and SLE shared many common characteristics, including inflammatory infiltration, estrogen association, enhanced immune response, and affecting women of reproductive age 10. Several epidemiologic studies have shown a strong association between endometriosis and SLE 11-13. Patients with endometriosis had a higher prevalence of SLE, and chronic inflammation and autoimmune dysfunction may be involved in the development of endometriosis and cause morbidity as a precursor state to endometriosis 4, 14. However, the relationship between endometriosis and SLE has not been thoroughly investigated, and the common molecular characteristics and underlying mechanisms remain unclear.

In the present study, based on the GEO database, we screened differently expressed genes in endometriosis and SLE, and successfully constructed prediction signatures using LASSO logistic regression analysis, respectively. The nomogram, calibration curve, decision curve analyses, and external datasets were used to verify the performance of the predictive signatures. Subsequently, we identified four co-diagnostic genes (PMP22, QSOX1, REV3L, SP110) for endometriosis and SLE, and comprehensively analyzed their biological characteristics, including protein interactions, functional annotations, multifactorial regulatory network, immune infiltration, potential target drugs prediction, and expression validation, to deeply explore the potential common pathogenic mechanisms between the two diseases. The results of this study will provide a novel perspective and candidate therapeutic targets for prognosis forecasting and personalized treatment of endometriosis and SLE.

## Materials and Methods

### Data collection and processing

The expression profiles related to SLE and endometriosis were obtained from the GEO database (http://www.ncbi.nlm.nih.gov/geo). The detailed information and screen criteria of dataset were shown in Table S1. The original data were normalized using the ComBat function in the “sva” R package to remove batch effects in the discovery cohort.

### Identification of Differently Expressed Genes (DEGs) in SLE and endometriosis

DEGs were identified using the “limma” R package (p < 0.05, | log2Fold Change (FC) | > 0.585) and visualized with volcano plots and heatmaps by the “ggplot2” R packThe weighted gene co-expression network analysis (WGCNA) was used to obtain co-expressed gene modules of high biological significance to endometriosis by the "goodSamplesGenes" function of the R package, and the appropriate soft-thresholding parameter was ascertained by the pickSoftThreshold function. The VENN diagram was applied to identified and visualized co- expression DEGs between SLE and endometriosis.

### Functional enrichment analysis

Kyoto Encyclopedia of Genes and Genomes (KEGG) and Gene Ontology (GO) enrichment analysis of the overlap DEGs were detected by the “ClusterProfiler”, “ggplot2”, and “pathview” R package (p < 0.05). The protein-protein interaction (PPI) network of overlap DEGs were construction by the STRING database (https://string-db.org) and visualized using Cytoscape (V3.10.1). The genomic enrichment analysis (GSEA) to assess variations in biological processes of diagnostic genes.

### Screening diagnostic genes using machine learning

The 15 genes and 6 genes were identified by Least Absolute Shrinkage and Selection Operator (LASSO) logistic regression analysis of SLE and endometriosis, respectively. The regression model was constructed using the “glmnet” R package and the optimal values of the penalty parameter λ were detected by 10-fold cross-validation. Four overlapping genes were identified as co-diagnostic genes for SLE and endometriosis. The nomogram was created by the “RMS” R package in SLE to score predictors and achieve patient stratification. The predictive performance of the nomogram was assessed using the calibration curve and decision curve analyses (DCA). Consensus clustering analysis was constructed using the “ConsensusClusterPlus” R package base on expression profiles of four diagnostic genes in endometriosis. The area under the receiver operating characteristic (ROC) curve (AUC) was performed to estimate the capability of diagnostic genes in SLE and endometriosis. The external datasets were utilized to verify the accuracy of diagnostic genes.

### Immune infiltration and correlation analysis

The infiltration of 22 immune cell subtypes in SLE and endometriosis were calculated using the CIBERSORT algorithm. The ESTIMATE algorithm was used to assess the ESTIMATE score, immune score, stromal score. Immune-related pathways were analyzed using the "GSVA" R package based on c7.immunesigdb.v7.4.symbols.gmt. We also investigated the correlation between immune cell fractions and diagnostic genes expressions.

### Transcription Factors (TF) - CeRNA network construction and potential drug prediction

Using the hTFtarget database (http://bioinfo.life.hust.edu.cn/hTFtarget#!/), we screened predictive TFs for diagnostic genes in SLE and endometriosis (screening criteria: tissues is “Blood” or “Peripheral blood” for SLE; tissues is “Endometrium” or “Uterus” for endometriosis). We used the miRWalk database (http://mirwalk.umm.uni-heidelberg.de/) to predict target miRNA and lncRNA paired with four diagnostic genes (screening criteria: CLIP-DATA≥1, Degradome-Data≥1). The potential drugs and inhibitors were predicted using the Coremine Medical database (https://coremine.com/medical/") (screening criteria: p <0.05). The network was visualized by Cytoscape (V3.10.1) and Autodock Vina was used to construct molecular docking between potential drug and four diagnostic genes.

### RNA extraction and real-time quantitative polymerase chain reaction (RT-qPCR)

Total RNA was isolated from normal endometrial of diseases-free women (N=10) and ovarian endometriotic tissues from endometriosis patients (N=10) using Trizol (RNAiso Plus; Takara, Japan) and reverse transcribed using PrimeScript™ RT reagent kit (RK20429; ABclonal, Wuhan, China). Following the manufacturer's instructions, RT-qPCR was carried out by a SYBR Green qPCR Supermix kit (RK12106, Abclonal, Wuhan, China). The relative expression levels of the diagnostic genes were normalized by β-actin and calculated using the 2-ΔΔCT method. The primer sequences were listed in Supplementary Table 2. The study was authorized by the Medical Ethics Committee of Shanghai First Maternity and Infant Health Hospital (KS21198), and each patient signed a written informed consent.

### Cell culture and in vitro experiments

The human endometrial stromal cell (hESCs) was cultured in DMEM-F12 (Servicebio, Wuhan, China) supplemented with 10% fetal bovine serum (Gibco, USA) and 1% penicillin/streptomycin (NCM, Suzhou, China) at 37 °C with 5% CO2. Small interfering RNAs (siRNAs) were purchased from Tsingke Biotech Co., Ltd (Beijing, China) and transfected by Lipofectamine 3000 (Invitrogen, USA). The western blot assay was performed as previously described 15, 16, and the antibodies were shown as follows: PMP22 (1:1000, A15083, ABclonal), SP110 (1:1000, A7492, ABclonal), β-actin (1:5000, 60008, proteintech) and secondary antibodies (1:3000, GB23204; Servicebio, Wuhan, China). CCK-8 and colony formation assay were detected to assess cell proliferation, while transwell and wound healing experiments were used to measure cell migration and invasion 17, 18.

### Statistical analysis

Statistical analysis was performed using R version 4.0.2 and visualized using Adobe Illustrator (version 27.0). The unpaired t-test and Wilcoxon Rank-Sum Test was used to assess the differences between the two groups. Pearson's correlation was used to detected correlations between variables. All statistical analyses were two-sided and p < 0.05 indicated statistically significant.

## Results

### Data processing and co-expression DEGs identification

**Figure 1:**
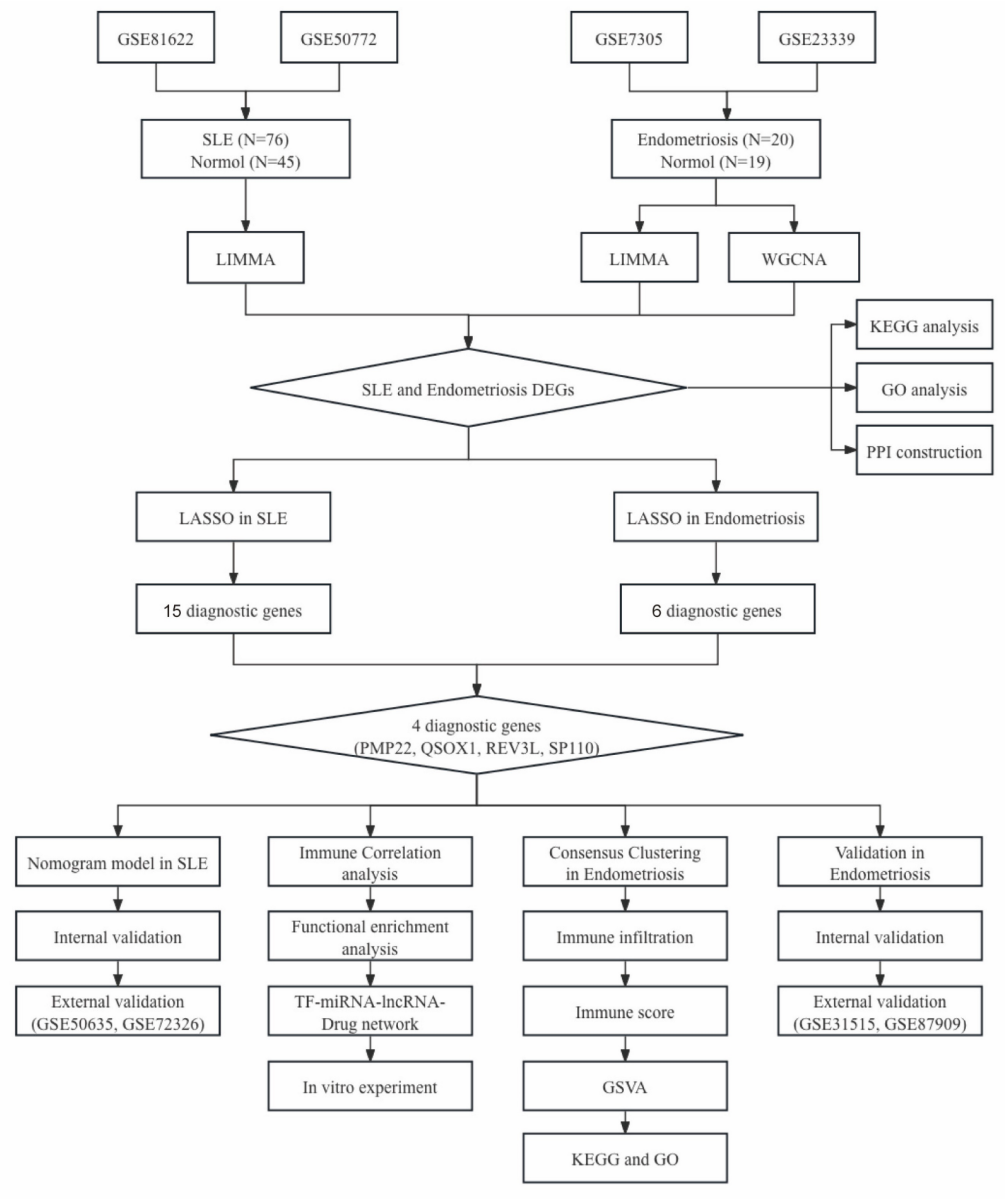
The flowchart of this study.

**Figure 2:**
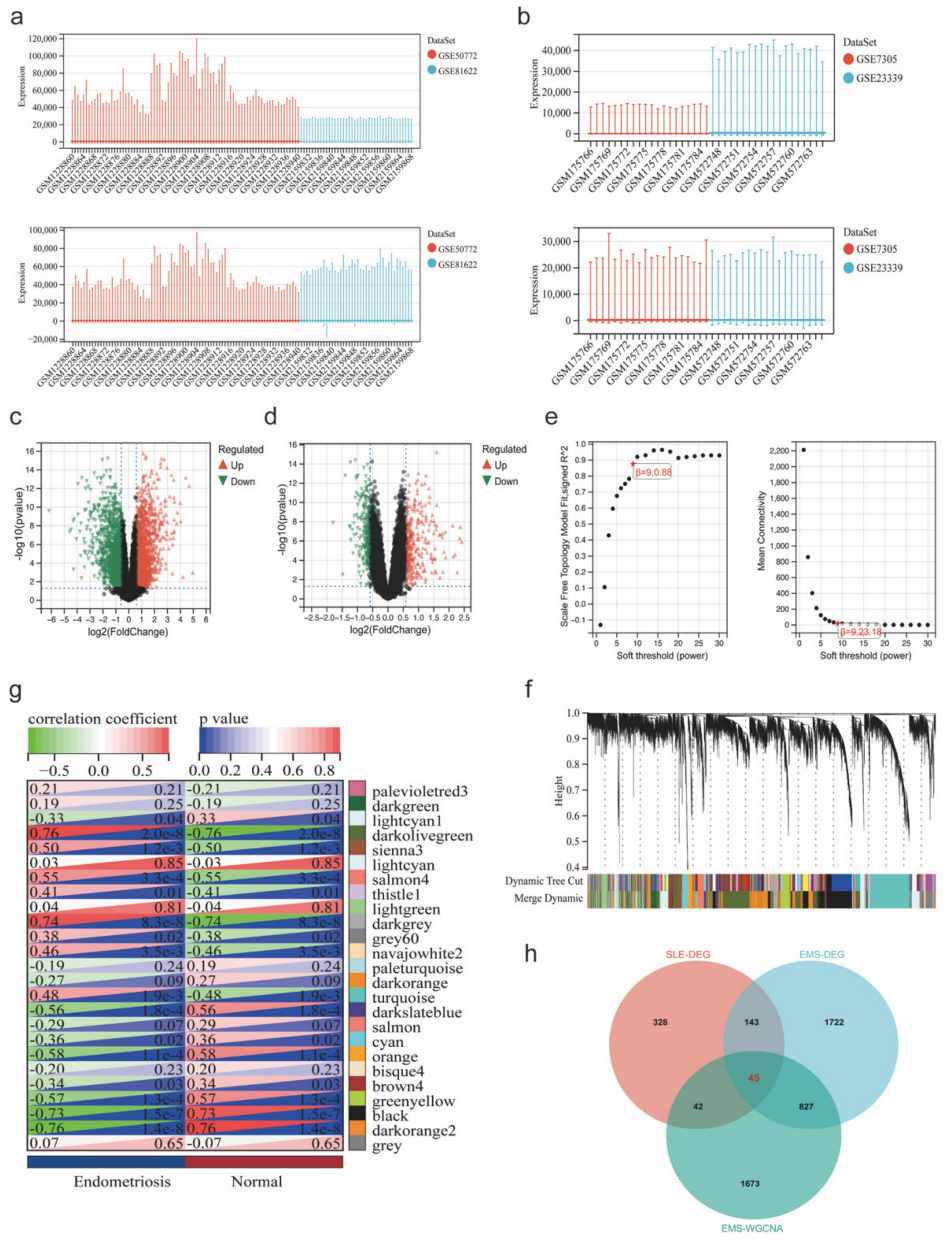
Identification of co-expression DEGs in the endometriosis and SLE cohort. (a, b) Expression distribution of the training cohorts for SLE and endometriosis before and after the elimination of batch effect. (c, d) Volcano plot of DEGs in the SLE and endometriosis training cohort. (e, f) Soft threshold selection and dynamic dendrogram of WGCNA analysis. (g) Module-trait heatmap demonstrated the relationship of module and trail. (h) Venn plot showed the overlapping genes of SLE and endometriosis.

The workflow of our study is shown in Figure 1. We combined GSE81622 and GSE50772 into a merged cohort of SLE (including 76 SLE samples and 45 normal samples), while merging GSE7305 and GSE23339 into an entire cohort for endometriosis (including 20 endometriosis samples and 19 normal samples) (Figure 2a, b). The density, and distribution of the datasets indicated a successful elimination of the batch effect (Figure S1a-d). Subsequently, we used the “limma” R package to identify DEGs between normal and disease group in SLE and endometriosis, respectively. A total of 558 DEGs were extracted in the SLE training cohort, with 306 upregulated and 252 downregulated genes (Figure 2c, Figure S1e). Meanwhile, the volcano plot (Figure 2d) and heatmap (Figure S1f) also showed that there were 2767 DEGs in the endometriosis training cohort, of which 1370 were significantly upregulated and 1367 were significantly downregulated. Besides, WGCNA was used to select significant module genes of endometriosis. The ideal soft threshold power (β) was identified 9 (R2 = 0.88) to build a scale-free topological network (Figure 2e, f). The darkolivegreen demonstrated the most significant positive correlation with endometriosis (r = 0.76, p = 2.0e-8), while the darkorange2 was the most negatively correlated module (r = -0.76, p = 1.4e-8) (Figure 2g). The correlation between gene significance and module membership in darkolivegreen (r = 0.71, p = 1.3e-252) and darkorange2 (r = 0.74, p = 4.0e-165) were shown in Supplementary Figure 1g, h. We screened 2105 significant module genes from the two modules. As shown in the Venn plot (Figure 1h), the intersection of DEGs of SLE, DEGs of endometriosis and module genes regarding endometriosis produced 45 overlapping genes related to both SLE and endometriosis.

### Functional enrichment analysis of overlapping genes

**Figure 3:**
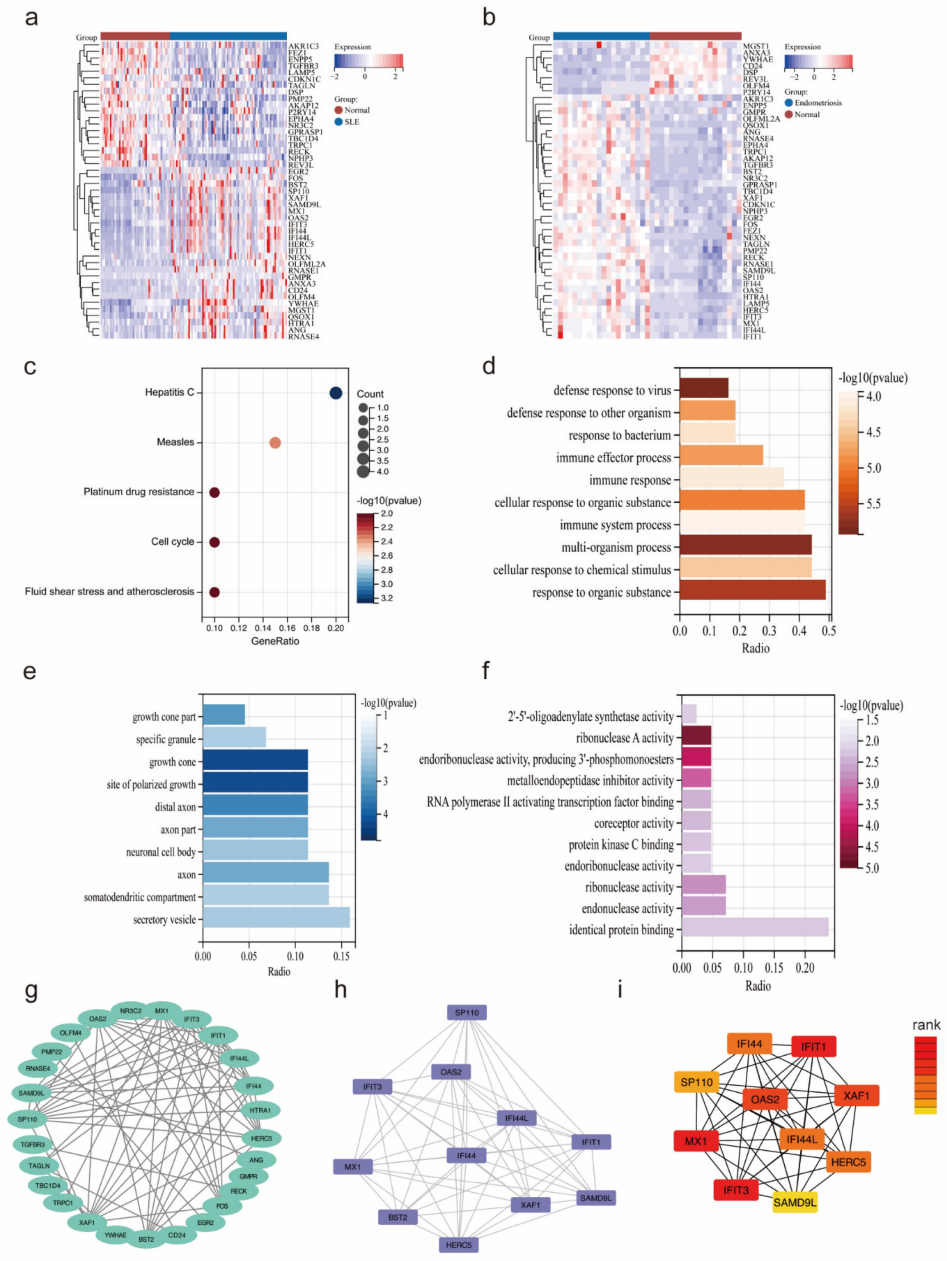
Functional enrichment analysis of overlapping genes. (a, b) The expression of 45 overlapping genes in the SLE and endometriosis cohort. (c) KEGG analysis of 45 overlapping genes. (d-f) GO analysis of 45 overlapping genes. (g-h) PPI network, key module, and hub genes of 45 overlapping genes.

The heatmaps showed the expression of 45 overlapping genes in the SLE training cohort (Figure 3a) and the endometriosis training cohort (Figure 3b). Subsequently, functional enrichment analysis was utilized to reveal the pathways in which they might be involved. The KEGG analysis showed that these overlapping genes were significantly enriched in measles, hepatitis, platinum drug resistance, cell cycle, and fluid shear stress and atherosclerosis (Figure 3c). GO analysis demonstrated a significant enrichment in biological process (BP), such as defense response to virus, multi-organism process and response to organic substance, as well as in cellular component (CC), including growth cone, site of polarized growth, and secretory vesicle and in molecular function (MF) including ribonuclease A activity, endoribonuclease activity, producing 3-phosphomonoesters and metalloendopeptidase inhibitor activity (Figure 3d-f). Besides, the PPI network was constructed with 27 nodes and 72 edges (Figure 3g). The key module was selected by MCODE (Figure 3h), and the top 10 hub genes were identified using cytoHubba (Figure 3i).

### Identification of potential co-diagnostic genes

**Figure 4:**
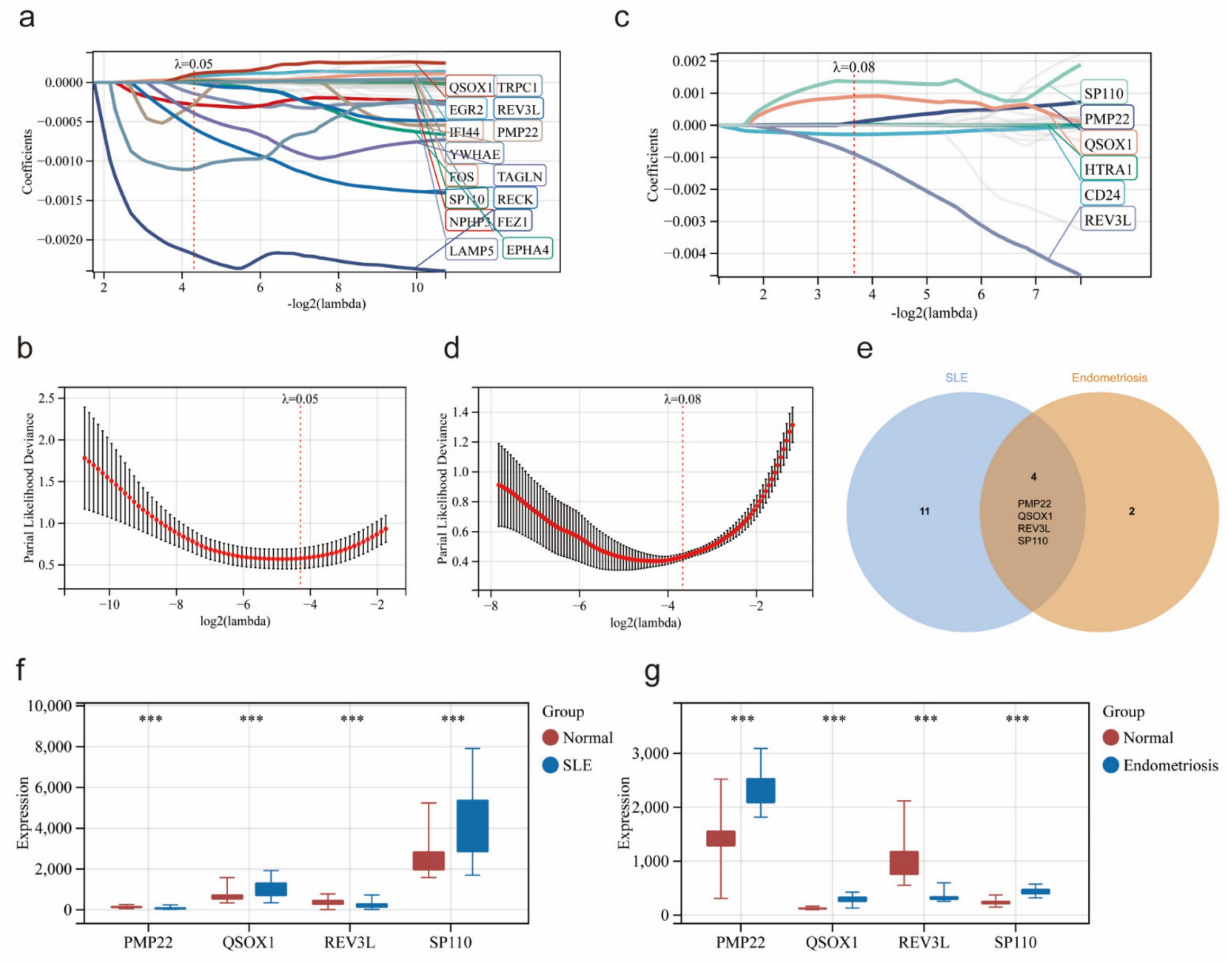
Identification of potential co-diagnostic genes. (a, b) Coefficient profiles and cross-validation of parameter of the LASSO regression in the SLE training cohort. (c, d) Coefficient profiles and cross-validation of parameter of the LASSO regression in the endometriosis training cohort. (e) Venn plot demonstrated the four co-diagnostic genes. (f-g) The expression of four co-diagnostic genes in the SLE and endometriosis cohort. * p<0.05, **p<0.01, ***p<0.001.

Based on 45 overlapping genes, the Lasso regression analysis was applied to further identify the most candidate co-diagnostic genes in SLE and endometriosis. In the SLE training cohort, the lambda value of 0.508 was regarded as the optimal lambda to screen the potential diagnostic genes, and 15 genes were identified (Figure 4a, b). Meanwhile, lambda was set at 0.788 based on the coefficient profiles and the optimal tuning parameter selection map of LASSO regression analysis in the endometriosis training cohort, and 6 genes were selected (Figure 4c, d). Subsequently, by the intersection analysis, we obtained four overlapping genes (PMP22, QSOX1, REV3L, SP110) as potential co-diagnostic genes for SLE and endometriosis (Figure 4e). We further analyzed the expression of four potential co-diagnostic genes in SLE and endometriosis, the results showed that QSOX1 and SP110 were significantly upregulated in both SLE and endometriosis group than in the normal group, and REV3L was significantly downregulated in both SLE and endometriosis group, while the expression of PMP22 was opposite in the SLE group (downregulated compared to the normal group), and endometriosis group (upregulated compared to the normal group) (Figure 4f, g).

### Functional enrichment analysis of potential co-diagnostic genes

**Figure 5:**
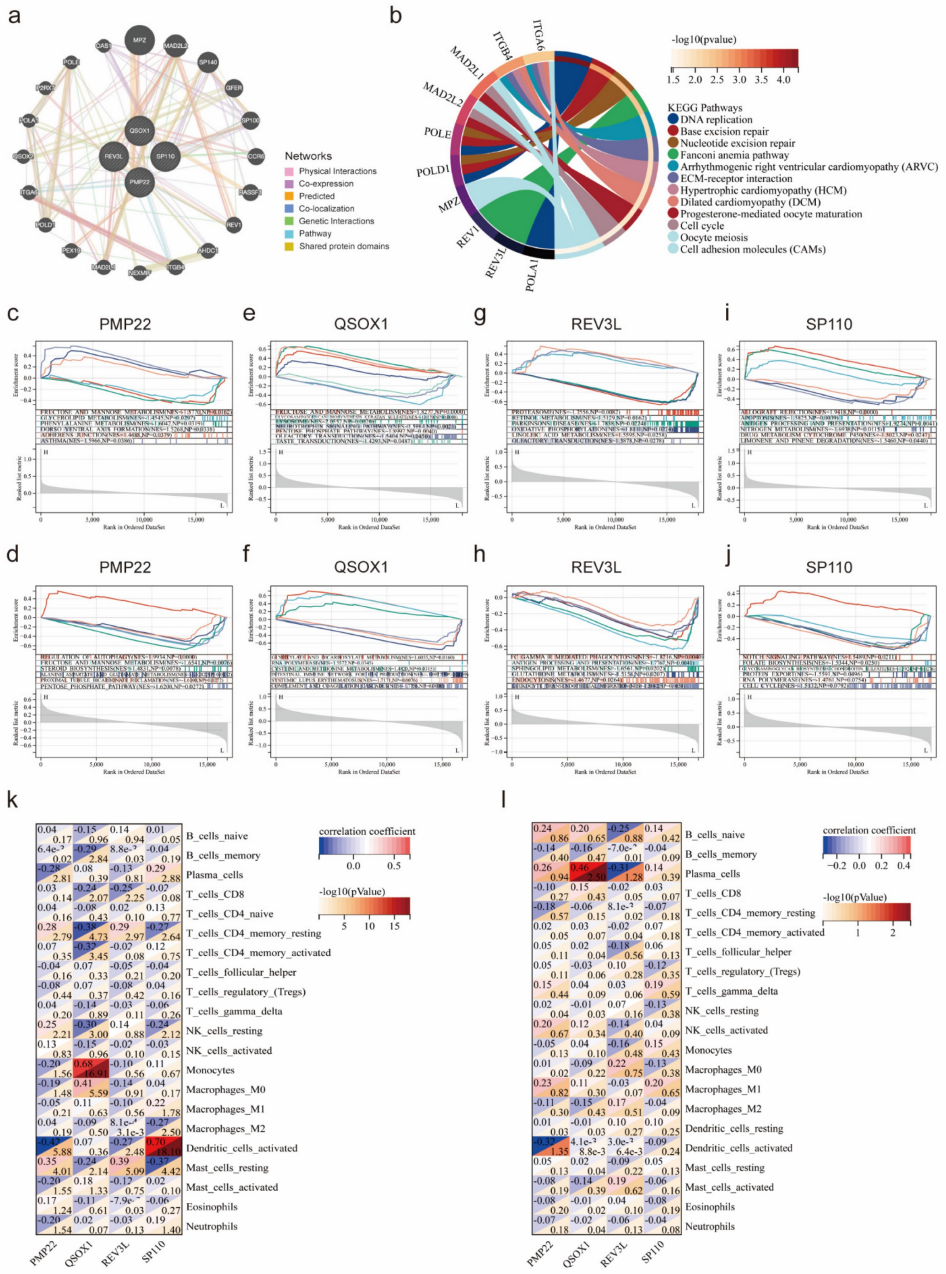
Functional enrichment analysis of potential co-diagnostic genes. (a) The co-expression network of four co-diagnostic genes. (b) KEGG analysis of co-expression genes. (c, e, g, i) GSEA of four co-diagnostic genes (PMP22, QSOX1, REV3L, SP110) in the SLE training cohort. (d, f, h, j) GSEA of four co-diagnostic genes (PMP22, QSOX1, REV3L, SP110) in the endometriosis training cohort. (k, l) The relationship of our potential co-diagnostic genes and immune cell infiltration in the SLE and endometriosis cohort.

The GeneMANIA database was applied to identify the 20 genes most associated with four potential co-diagnostic genes and constructed an interaction network (Figure 5a). Subsequently, we analyzed the functions of 24 genes by the KEGG analysis, and the result demonstrated that DNA replication, base excision repair, cell cycle and cell adhesion molecules (CAMs) were significantly enriched (Figure 5b). We also utilized a GSEA method to further explore the function of four potential co-diagnostic genes in SLE and endometriosis, respectively. In both SLE and endometriosis, PMP22 was highly enriched in FRUCTOSE_AND_MANNOSE_METABOLISM (NES = -1.5770, NP = 0.0162; NES = 1.9934, NP = 0.000) (Figure 5c, d). QSOX1 was also enriched in FRUCTOSE_AND_MANNOSE_METABOLISM (NES = 1.8277, NP = 0.0000) in SLE, while mainly involved in GLYOXYLATE_AND_DICARBOXYLATE_METABOLISM (NES = 1.6055, NP = 0.0160) in endometriosis (Figure 5e, f). For REV3L, the PROTEASOME pathway (NES = -1.7556, NP = 0.0082) was enriched in SLE, and FC_GAMMA R_MEDIATED_PHAGOCYTOSIS pathway (NES = - 1.8216, NP = 0.0040) was enriched in endometriosis (Figure 5g, h). Meanwhile, SP110 was mostly engaged in ALLOGRAFT REJECTION (NES = 1.9418, NP = 0.0000) in SLE, and enriched in NOTCH SIGNALING PATHWAY (NES = 1.5489, NP = 0.0211) in endometriosis (Figure 5i, j). Furthermore, we found a high correlation between four potential co-diagnostic genes and immune cell infiltration in SLE and endometriosis, such as QSOX1 was highly related to Monocytes (cor = 0.68, -log 10 (p value) = 16.91) in SLE, while strongly associated with Plasma cells (cor = 0.46, -log 10 (p value) = 2.50) in endometriosis (Figure 5k, l).

### Construction and validation of a nomogram in SLE

**Figure 6:**
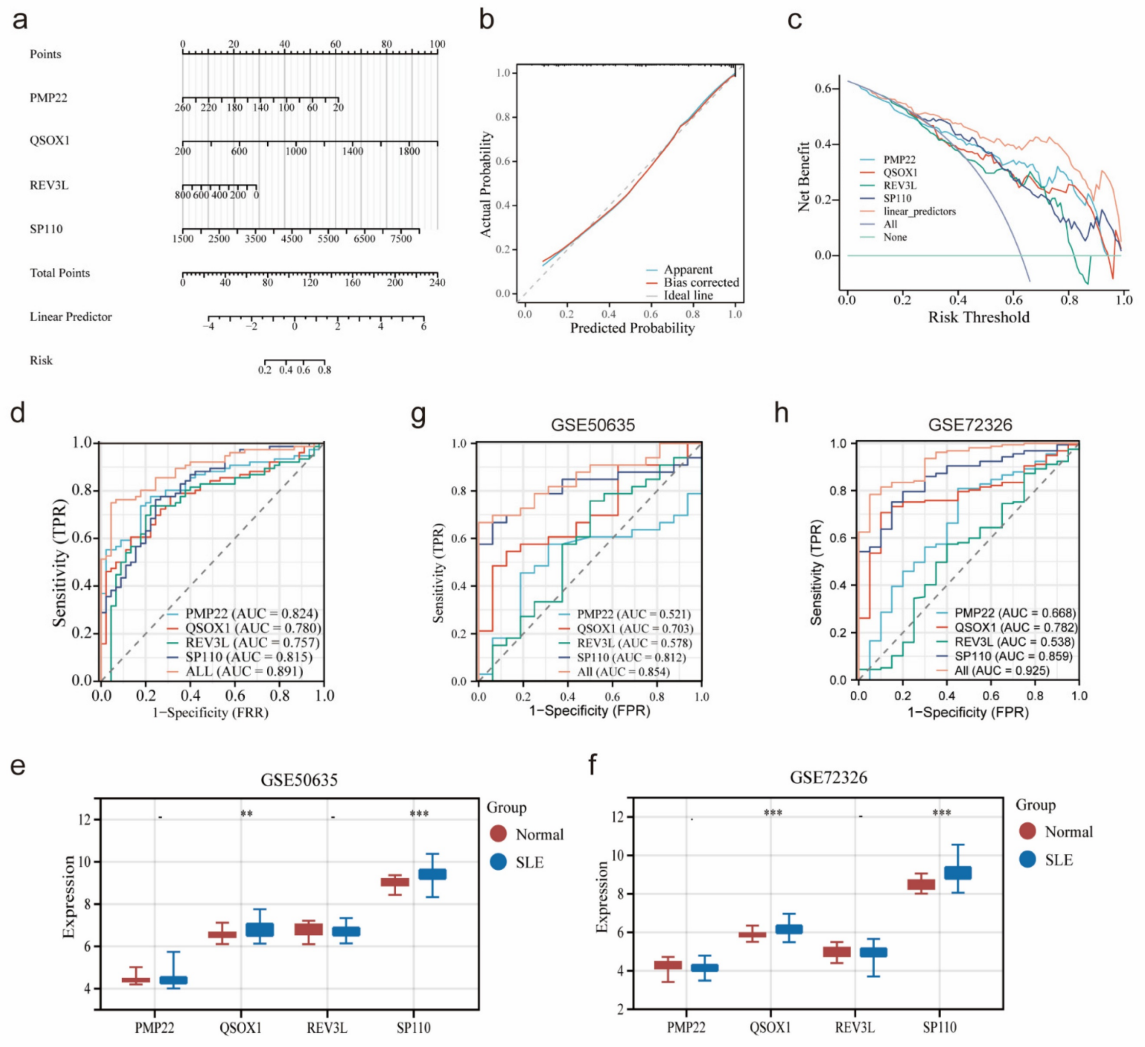
Construction and validation of a nomogram in SLE. (a) Nomogram based on four co-diagnostic genes in the SLE training cohort. (b-d) The calibration curve, DCA and ROC curve of diagnostic model in the SLE training cohort. (e, f) Expression of four co-diagnostic genes in the SLE validation cohorts (GSE60635 and GSE72326). (g, h) ROC curve of diagnostic model in the SLE validation cohorts (GSE60635 and GSE72326). * p<0.05, **p<0.01, ***p<0.001.

Based on the expression of four potential co-diagnostic genes, a diagnostic model was constructed to assess the predictive efficiency by nomogram (Figure 6a). The calibration curve and DCA revealed that the nomogram had high accuracy for diagnosing endometriosis (Figure 6b, c). Meanwhile, the ROC curve demonstrated that the four diagnostic genes prediction model had a satisfactory capability to differentiate between SLE patients and normal cases, with an AUC value of 0.891, which was higher than that of an individual diagnostic gene (Figure 6d). Besides, two external cohorts (GSE50635 and GSE72326) were used to validate the accuracy of the diagnostic model. The expression of four diagnostic genes were examined and the result showed that the expression patterns of QSOX1 and SP110 were consistent with those in the SLE training cohort (Figure 6e, f). Furthermore, the AUC value of diagnostic model in GSE50635 and GSE72326 were also high, at 0.854 and 0.925, respectively (Figure 6g, h). These results indicated that four diagnostic genes had high efficiency in diagnosing SLE.

### Validation diagnostic genes and identification subtypes in endometriosis

**Figure 7:**
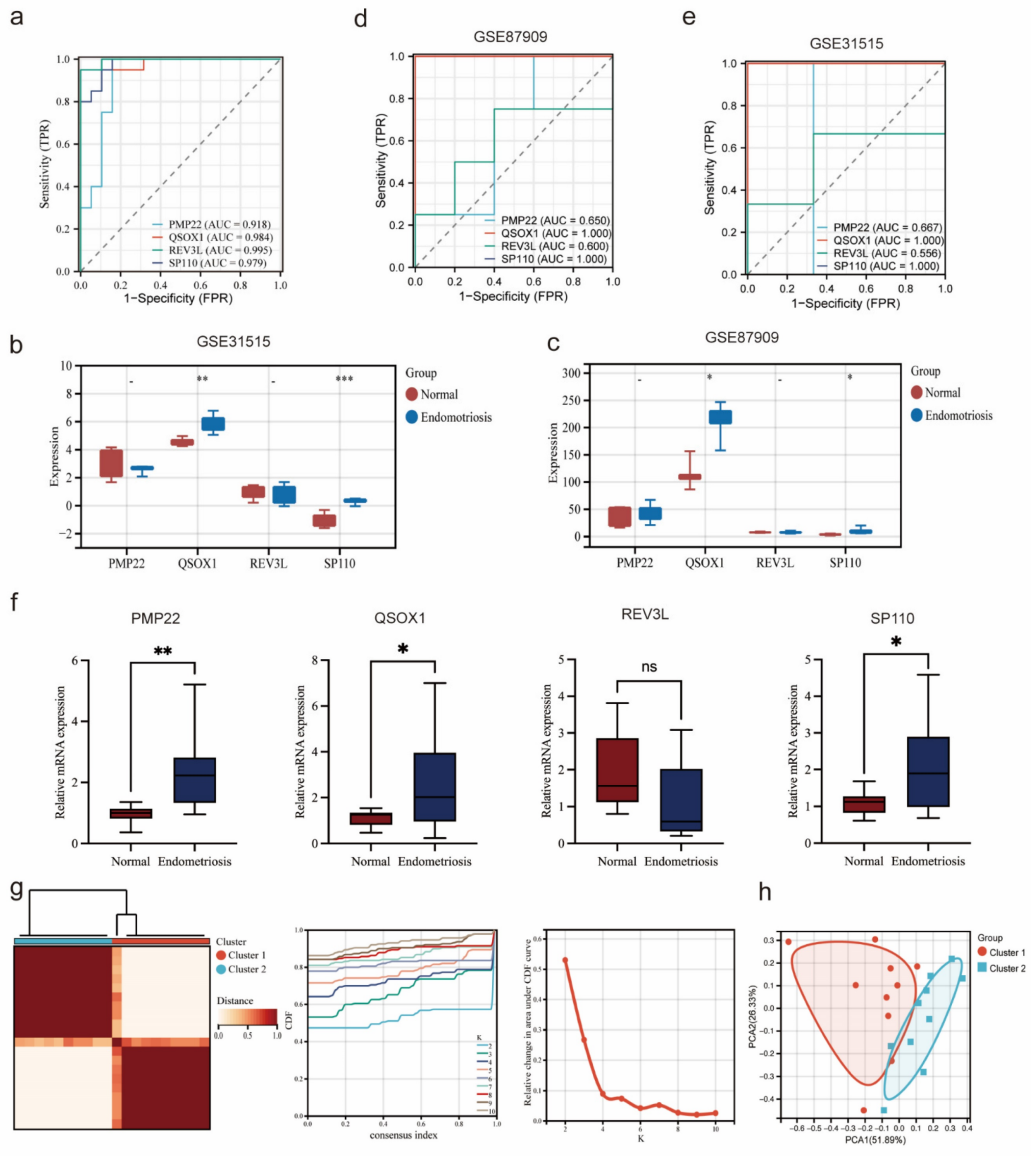
Validation diagnostic genes and identification subtypes in endometriosis. (a) ROC curve of four co-diagnostic genes in the endometriosis training cohort. (b, c) Expression of four co-diagnostic genes in the endometriosis validation cohorts (GSE31515 and GSE87909). (d, e) ROC curve of our co-diagnostic genes in the endometriosis validation cohorts (GSE31515 and GSE87909). (f) Expression of four co-diagnostic genes in the clinical samples. (g) The consensus clustering matrix (k = 2), consensus distribution function (CDF), and delta area of consensus clustering analysis based on endometriosis samples in the endometriosis training cohort. (h) The PCA diagram assessed the stability and reliability of the clustering analysis. * p<0.05, **p<0.01, ***p<0.001.

As shown in the Figure 7a, the AUC value of four potential co-diagnostic genes were higher than 0.918 in the endometriosis training cohort. The expression of four potential co-diagnostic genes in the endometriosis validation cohort (GSE31515 and GSE87909) showed that the expression of QSOX1 and SP110 were significantly upregulated in the endometriosis group than normal group, which were consistent with the training group (Figure 7b, c). Besides, the AUC value was also performed to assess the sensitivity and specificity of four potential co-diagnostic genes in the validation cohort. The results showed that QSOX1 and SP110 had strong predictive ability and their AUC values were also higher than 0.900 (Figure 7d, e). To further evaluate the diagnostic value of four potential co-diagnostic genes, we examined the expression levels in clinical tissues, including normal endometrial of diseases-free women and ovarian endometriotic tissues from endometriosis patients. The expression of PMP22, QSOX1 and SP110 were significantly higher in the ovarian endometriotic tissues from endometriosis patients compared to normal endometrial. REV3L was not statistically significant, but its expression trend was consistent with that observed in the endometriosis training cohort (Figure 7f). Subsequently, according to the expression of four potential co-diagnostic genes, we clustered endometriosis samples in the endometriosis training cohort using consensus clustering analysis. When the consensus matrix was k = 2, the classification demonstrated high reliability and stability (Figure 7g), and the PCA showed a significant difference between the two subtypes (Figure 7h).

### Immune infiltration and correlation analysis of endometriosis subtypes

**Figure 8:**
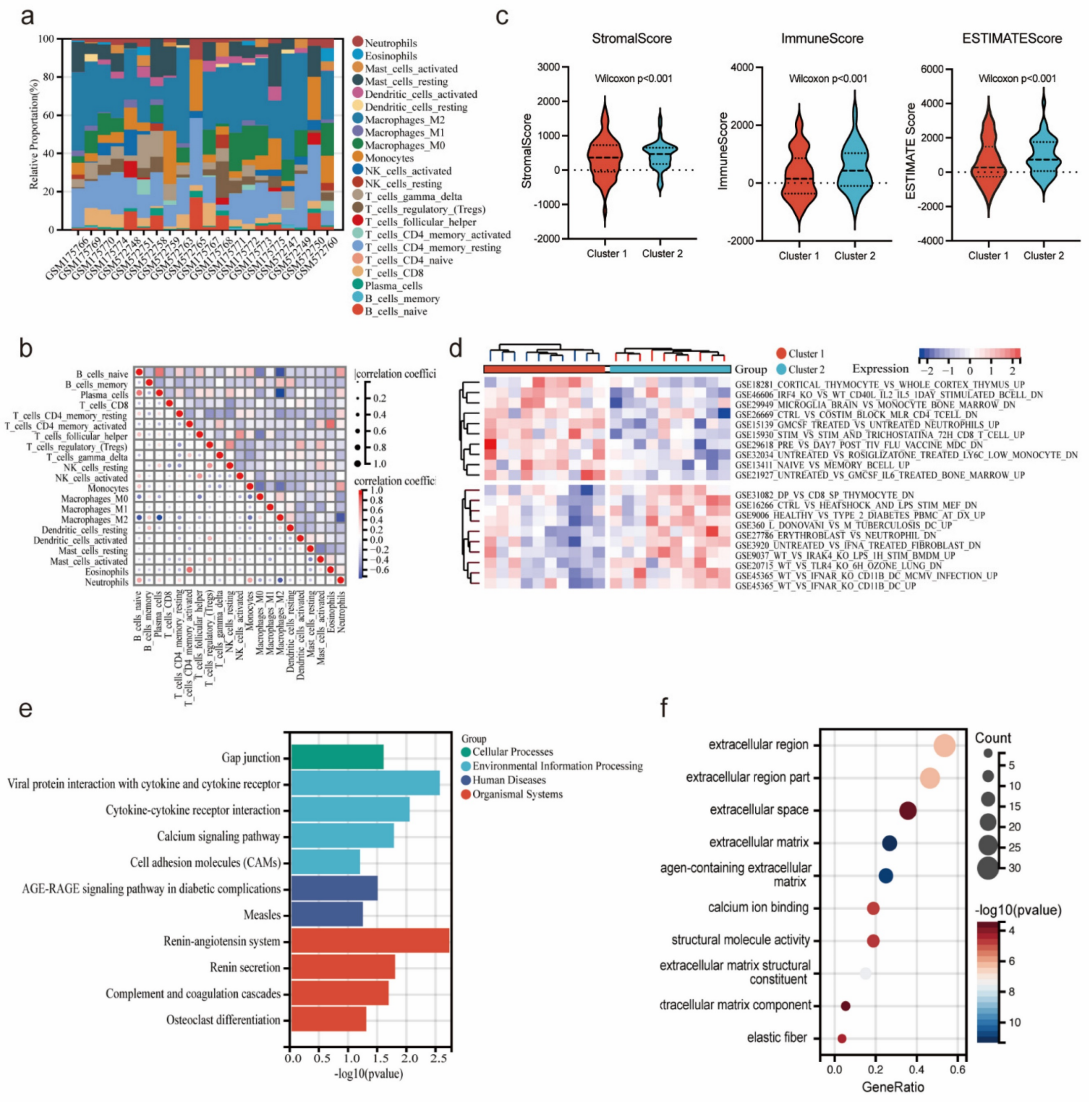
Immune infiltration and correlation analysis of endometriosis subtypes. (a) Stacked Bar Chart depicted the abundance immune cells of each endometriosis samples. (b) The correlation of immune cells. (c) Stromal Score, immune score, and ESTIMATE score between the two clusters (Wilcoxon p<0.001). (d) The heatmap displayed the result of GSVA between the two clusters. (e,f) KEGG and GO analysis of DEGs between the two clusters.

Based on the infiltration of immune cells determined by the CIBERSORT algorithm, the immune cell composition abundance of endometriosis subtype samples was demonstrated in Figure 8a, indicating that immune factors may influence the progression of two endometriosis subtypes. And correlation analysis between immune cell types showed a positive correlation between T cell regulatory (Tregs) and NK cell resting in endometriosis patients (cor = 0.70, p < 0.05) (Figure 8b). We also employed the ESTIMATE algorithm to calculate the stromal score, immune score, and ESTIMATE score for the endometriosis subtypes. The results showed that the cluster 2 had a significantly higher stromal score, immune score, and ESTIMATE score than the cluster 1 (Figure 8c). And we analyzed the immune-related pathways by GSVA algorithm. The heatmap depicted that cluster 1 showed an active activity in GSE18281_CORTICAL_THYMOCYTE_VS_WHOLE_CORTEX_THYMUS_UP, and cluster 2 was significantly enriched in GSE31082_DP_VS_CD8_SP_THYMOCYTE_DN (Figure 8d). Additionally, we identified DEGs between the endometriosis subtypes and constructed enrichment analysis. KEGG analysis demonstrated that DEGs between the endometriosis subtypes were enriched in gap junction, viral protein integration with cytokine and cytokine receptor and renin-angiotensin system (Figure 8e). GO analysis showed that DEGs between the endometriosis subtypes were actively gathered in extracellular region, extracellular region part and extracellular space (Figure 8f). These results suggested that endometriosis subtypes based on four diagnostic genes were important for the diagnosis of endometriosis.

### Construction of TFs-ceRNA-drug network of potential co-diagnostic genes

**Figure 9:**
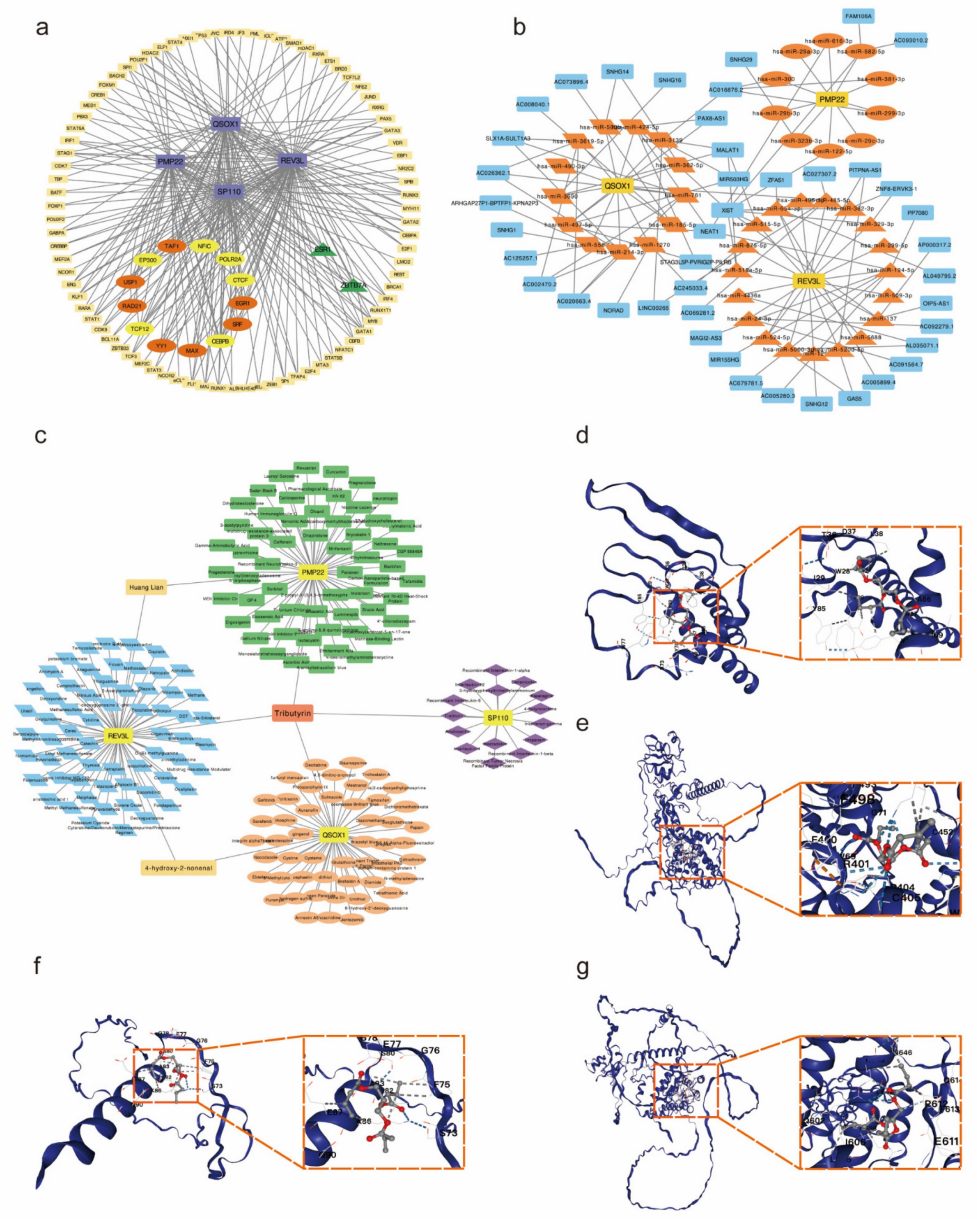
Construction of TFs-ceRNA-drug network of potential co-diagnostic genes. (a) The TF-diagnostic genes network. (b) The ceRNA-diagnostic genes network. The yellow represents co-diagnostic genes. The tangerine represents miRNAs. The blue represents lncRNAs. (c) The drug-dagnostic genes network. (d-g) Molecular docking analysis of Tributyrin and four potential co-diagnostic genes (PMP22, QSOX1, REV3L, SP110).

To explore the potential regulatory interactions, we establish a multifactorial interaction network consisting of TFs, miRNA, lncRNA and drugs associated with potential co-diagnostic genes. We screened 96 TFs related to four potential co-diagnostic genes in SLE, and 15 TFs in endometriosis. The results indicated that 13 TFs were identified in both SLE and endometriosis, and 6 of them (EP300, TCF12, CTCF, POLR2A, CEBPB and NFIC) can act on four potential co-diagnostic genes simultaneously (Figure 9a). The ceRNA network described mutual integration between four potential co-diagnostic genes and non-coding transcripts (including miRNAs and lncRNAs). A total of 42 miRNA and 43 lncRNA were screened and a ceRNA network with 88 nodes and 120 edges was constructed (Figure 9b). Additionally, we predicted drugs and inhibitors and extracted drug- marker interactions based on four potential co-diagnostic genes using the Coremine Medical database. And a drug- marker network containing 193 nodes (including 4 potential co-diagnostic genes and 189 drugs) and 194 edges was established. The result demonstrated that Huang Lian could act on PMP22 and REV3L, 4-hydroxy-2-nonenal could act on QSOX1 and REV3L, and Tributyrin could simultaneously act on the four potential co-diagnostic genes (Figure 9c). And the molecular docking between Tributyrin and four potential co-diagnostic genes was constructed (Figure 9d-g).

### Verification of potential co-diagnostic genes

**Figure 10:**
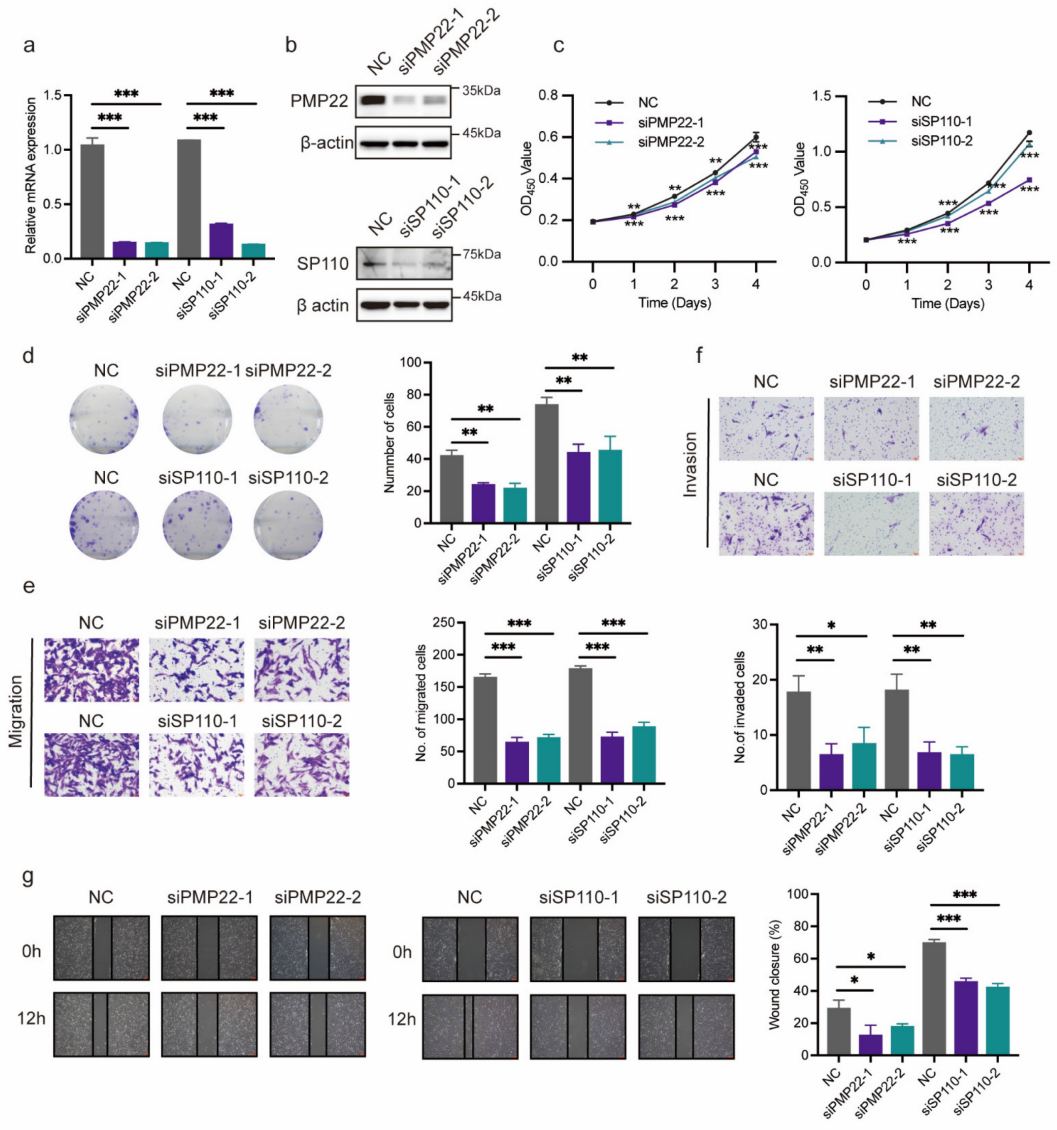
Verification of potential co-diagnostic genes. (a, b) The efficiency of PMP22 and SP110 knockdown by qPCR and western blot. (c, d) The CCK-8 and colony formation assay assessed the proliferation of hESCs after PMP22 and SP110 knockdown. (e-g) The migration and invasion abilities of hESCs after PMP22 and SP110 knockdown were evaluated by the transwell and wound healing assays. * p<0.05, **p<0.01, ***p<0.001.

PMP22 and SP110 were selected for in vitro experiments to further evaluate the function of potential co-diagnostic genes. We first silenced PMP22 and SP110 in hESCs with siRNAs, respectively (Figure 10a, b). CCK-8 and colony formation assays showed that the knockdown of PMP22 and SP110 significantly decreased the proliferation of hESCs (Figure 10c, d). Additionally, PMP22 and SP110 knockdown also significantly inhibited the migration and invasion of hESCs in Transwell and wound healing experiments (Figure 10e-g). These results pointed to a potential role for PMP22 and SP110 in the development of endometriosis.

## Discussion

Endometriosis has a high prevalence, affecting approximately 200 million women worldwide, with 50-80% of women suffering from pelvic pain and up to 50% of women expiring infertility 19, 20. Given the inadequate of disease recognition, 65% of women are initially misdiagnosed, with the diagnosis time from 4 to 11 years 21. Patients with endometriosis have a significantly lower quality of life and endure enormous financial burden 22. As a systemic autoimmune disease, the prevalence of SLE increases over time and primarily affects young women, resulting in decreased ovarian function and poor reproductive outcomes 23. Currently, medications and multidisciplinary treatments for SLE can only control symptoms and slow progression, and do not provide a complete cure 24. Importantly, endometriosis and SLE have several similar characteristics, such as chronic inflammation, immunological abnormalities, estrogen predominance 5. Patients usually have a poor prognosis with severe decreases in fertility and quality of life due to delayed diagnosis and limited response to current treatment for endometriosis and SLE 25, 26. Therefore, early detection and prevention are essential for precise individualized treatment of endometriosis and SLE in women.

In this study, we first identified 45 genes that were differentially expressed in both endometriosis training cohort and SLE training cohort. Functional annotation analysis demonstrated that these genes have ribonuclease A, endonuclease and metalloendopeptidase inhibitor activities, exerting important roles in viral defense, response to multi-organism process and organic substance, as well as in processes such as measles, hepatitis, platinum drug resistance and the cell cycle. Subsequently, by LASSO regression analysis, we constructed prediction models for 15 diagnostic genes in the endometriosis cohort and 6 diagnostic genes in the SLE cohort, respectively, and identified four co-diagnostic genes (PMP22, QSOX1, REV3L, SP110). In the SLE training cohort, we performed nomogram using 4 co-diagnostic gene, and calibration curve, DCA, ROC curve (AUC = 0.891) and external datasets showed that the model had higher diagnostic efficiency. We similarly found that four co-diagnostic genes also performed satisfactory predictive abilities in the endometriosis training and validation cohort. Additionally, we clustered endometriosis samples into two subtypes based on four co-diagnostic genes, with significant differences in immune infiltration. Our study may provide new perspectives for identifying potential co-diagnostic biomarkers and exploring underlying molecular processes for both endometriosis and SLE.

Peripheral myelin protein 22 (PMP22), a 22-kD transmembrane glycoprotein, has reported to be a culprit gene for most hereditary neuropathies 27. Recent studies have shown that PMP22 is involved in the occurrence and progression of many cancers, such as gastric cancer, liver cancer and pancreatic cancer 28, 29. In this study, we found that PMP22 expression was significantly increased in endometriosis patients and decreased in SLE patients, which may be related to the complex post-transcriptional regulation of PMP22. Quiescin sulfhydryl oxidase (QSOX1), a disulfide catalyst localized mainly in the Golgi apparatus and intracellular vesicles, participated in various cancer-related processes by catalyzing disulfide bond formation and reducing oxygen to hydrogen peroxide during protein folding 30, 31. In hepatocellular carcinoma, QSOX1 impaired cellular antioxidant capacity and promotes sorafenib-induced ferroptosis 32. And QSOX1 promoted the invasion of pancreatic and breast cancer cells 33. Our study showed that QSOX1 was significantly upregulated in both endometriosis patients and SLE patients, which may be responsible for excessive oxidative stress in chronic inflammation and immunological disorders. The reversionless 3-like (REV3L) is the catalytic subunit of DNA polymerase ζ, which participates in DNA synthesis, and played a critical role in chemoresistance in a variety of cancers 34. Studies demonstrated that downregulated REV3L significantly increased the sensitivity of cancer cells (such as glioma, non-small cell lung cancer, cervical cancer) to cisplatin 35-37. In our research, REV3L was significantly downregulated in endometriosis patients and SLE patients, and primarily involved in the PROTEASOME and FC_GAMMA R_MEDIATED_PHAGOCYTOSIS pathway. SP110, an interferon-induced nuclear protein, exerts transcriptional regulation through gene polymorphisms and is strongly associated with tuberculosis susceptibility 38, 39. We found that SP110 was significantly upregulated in endometriosis patients and SLE patients, which may be related to the aberrant immune infiltration. Collectively, PMP22, QSOX1, REV3L, SP110 may serve as potential diagnostic biomarkers for early diagnosis and targeted therapy of endometriosis and SLE.

To further explore the role of four co-diagnostic genes in endometriosis and SLE, we investigated multifactorial interaction networks including TF-ceRNA regulation and potential target drugs. Tributyrin, a neutral short-chain fatty acid triglyceride, presented in some spice plants at low levels in nature 40. Several research reported that Tributyrin, also a butyric acid prodrug existed in milk fat and honey, exerted anti-tumor effects in various cancers by inhibiting proliferation, promoting apoptosis, and stimulating differentiation without affecting non-cancer cells 41, 42. Recent studies have found that Tributyrin also plays an essential role in resisting inflammation injury, improving ovarian function, alleviating gut microbiota dysbiosis, and reducing immune stress 43-45. In our study, Tributyrin was identified as targeting drug for four co-diagnostic genes and has great potential to the precise personalized treatment of endometriosis and SLE.

Although some population-based cohort studies have investigated the correlation between endometriosis and SLE, our study is the first to explore common diagnostic markers and pathogenic mechanisms between endometriosis and SLE based on bioinformatics and machine learning. However, there were several limitations in our study. First, our study was based on public databases with a small sample size for clinical validation. It is necessary to further expand the clinical sample as well as conduct large-scale prospective study to further assess the diagnostic performance of four co-diagnostic genes. Besides, we only performed clinical validation in normal endometrial tissues and ovarian endometriotic tissues. In the future, we plan to collect endometrial tissues from patients with SLE and ovarian endometriotic tissues from patients with both endometriosis and SLE to evaluate the expression level and diagnostic value of the four co-diagnostic genes. Second, further in vivo and in vitro experiments are required to investigate the protein expression levels and detailed mechanisms of the four co-diagnostic genes, which will help the clinical application of the co-diagnostic genes in the future. Finally, we preliminarily predicted potential drugs targeting the four co-diagnostic genes in endometriosis and SLE, and further extensive in vivo and in vitro drug investigations and clinical trials are needed to confirm our predictions.

## Conclusions

We identified and validated PMP22, QSOX1, REV3L, SP110 as potential co-diagnostic genes associated with endometriosis and SLE and revealed the common pathogenic mechanisms, analyzed immune infiltration, constructed multifactorial interaction networks, as well as screened potential targeted therapeutic drugs. Our study provides a new idea and molecular foundation for the early clinical diagnosis and personalized treatment of endometriosis and SLE patients.

## Supplementary Material

Supplementary figure and tables.
