# Supplementary material for: Identification of common diagnostic genes and molecular pathways in endometriosis and systemic lupus erythematosus by machine learning approach and in vitro experiment
Source: Int J Med Sci. 2025 Jan 1;22(1):27–43. doi: 10.7150/ijms.101754 (PMC11659837; doi:10.7150/ijms.101754)
Supplement: Supplementary file 1 — Supplementary figure and tables. [file ijmsv22p0027s1.pdf]

Table S1. Information of GEO datasets

GSE	Platform	Samples	Tissues	Group
GSE81622	GPL10558	15 SLE and 25 Normal	PBMC	Training cohort
GSE50772	GPL570	61 SLE and 20 Normal	PBMC	Training cohort
GSE50635	GPL6244	33 SLE and 16 Normal	WB	Validation cohort
GSE72326	GPL10558	157 SLE and 20 Normal	WB	Validation cohort
GSE7305	GPL570	10 EMS and 10 Normal	Ovarian (EMS) Endometrium (Normal)	Training cohort
GSE23339	GPL6102	10 EMS and 9 Normal	Ovarian (EMS) Endometrium (Normal)	Training cohort
GSE87809	GPL11154	4 EMS and 5 Normal	ESC of Ovarian (EMS) nESC (Normal)	Validation cohort
GSE31515	GPL6480	6 EMS and 6 Normal	ESC of Ovarian (EMS) nESC (Normal)	Validation cohort

SLE filtering criteria:

(a) Datasets containing SLE and normal samples, and sample size comparatively large;

(b) Sequencing tissues were derived from human PBMC or WB.

EMS filtering criteria:

(a) Sequencing tissues were derived from human ovarian endometriosis or normal endometrium;

(b) EMS patients and normal women is in the proliferative phase of the menstrual cycle;

(c) Nine or more samples.

GEO: Gene Expression Omnibus, SLE: Systemic lupus erythematosus, PBMC: Peripheral blood mononuclear cell, WB: Whole Blood, EMS: Endometriosis, ESC: Endometrial stromal cells, nESC: normal endometrial stromal cells.

Table S2. The sequences of primers

Genes	Forward-Sequence (5’-3’)	Reverse-Sequence (5’-3’)
PMP22	ATCGTCAGCCAATGGATCGTG	AGAAACAGTGGTGGACATTTCC
QSOX1	TGAGAAAGTTTGGTGTCACCG	GGACCTGGATTCCATGAGCAC
REV3L	AATGTGGCTTTAGGCAATCC	AGGTAGGGAATATGCGCTTCA
SP110	CCTATGCCATACACAAGCCATT	CCTCTCCAGTTGGGTGAGAAT

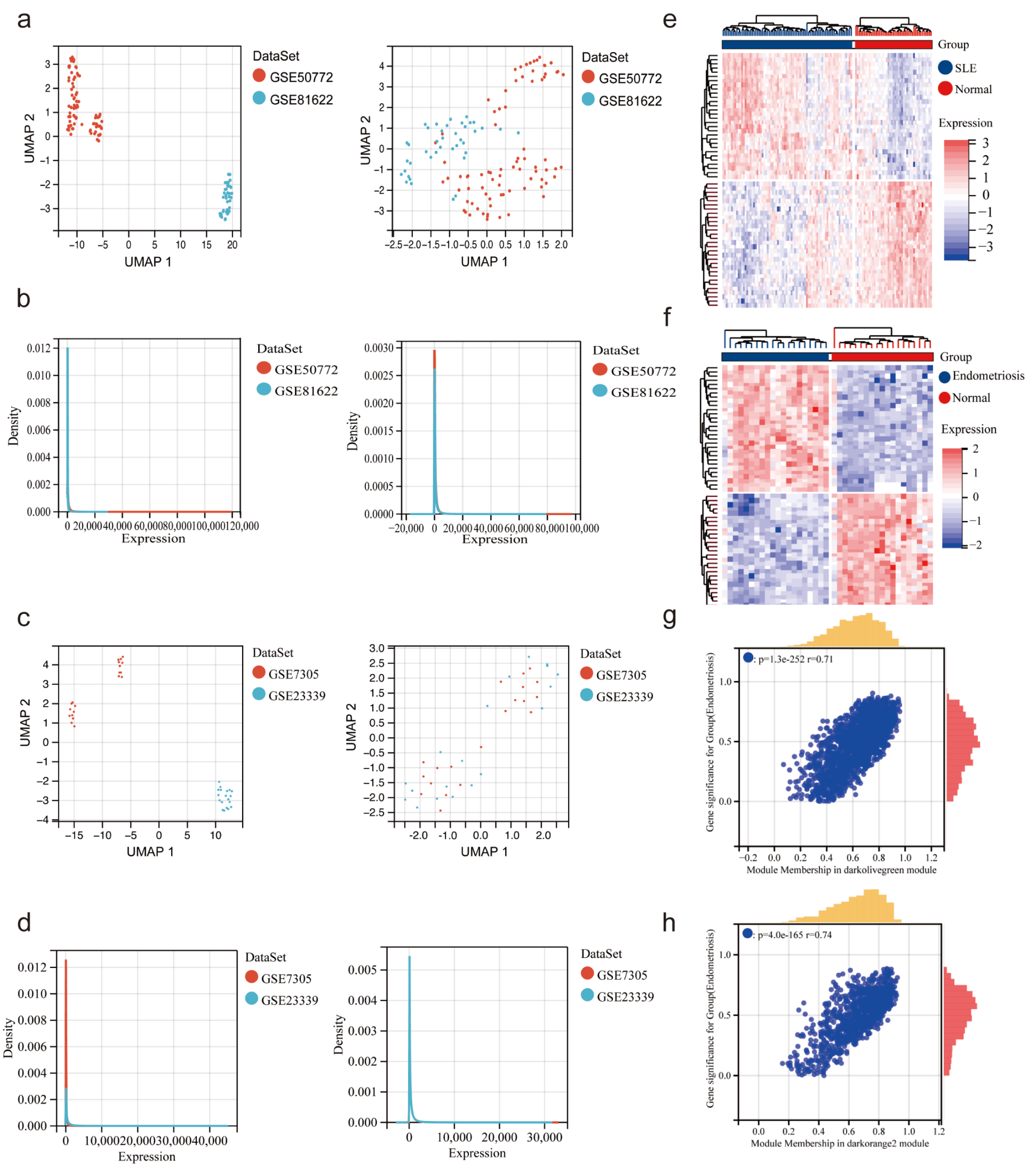

Figure S1. Data processing of endometriosis and SLE cohorts.

(**a, b**) The density and distribution of the SLE training cohort before and after the elimination of batch effect. (**c, d**) The density and distribution distributions of the endometriosis training cohort before and after the elimination of batch effect. (**e, f**) Heatmap of DEGs in the SLE and endometriosis training cohort. (**g, h**) The relationship between gene significance (GS) and module membership (MM) in darkolivegreen and darkorange2.
